# Supplementary material for: Characterization and functional analysis of the Hydroxycinnamoyl-CoA: shikimate hydroxycinnamoyl transferase (HCT) gene family in poplar
Source: PeerJ. 2021 Feb 25;9:e10741. doi: 10.7717/peerj.10741 (PMC7916539; doi:10.7717/peerj.10741)
Supplement: Supplemental Information 4 [file peerj-09-10741-s004.docx]

**Supplementary Table 4-1**HPLC gradient program for detecting caffeic acid, shikimate and caffeoyl shikimate

| Time（min） | Flow（ml/min） | acetonitrile（%） | 0.01% formic acid |
| --- | --- | --- | --- |
| 0 | 0.1 | 0 | 100 |
| 24 | 0.1 | 35 | 65 |
| 27 | 0.1 | 100 | 0 |
| 32 | 0.1 | 100 | 0 |
| 35 | 0.15 | 0 | 100 |
| 55 | 0.1 | 0 | 100 |

**Supplementary Table 4-1** Optimized MSn condition for caffeic acid, shikimate and caffeoyl shikimate

| compound | Scan model | Precursor  ion | Collision  energy (eV) (%) | Product ion  of SRM mode (MS_2_) |
| --- | --- | --- | --- | --- |
| caffeic acid | （-） | 180 | 35 | 143,135，106 |
| shikimate | （-） | 174 | 35 | 172,146,111 |
| caffeoyl shikimate | （-） | 336 | 35 | 179,161,135 |
