# Supplementary material for: Characterization and functional analysis of the Hydroxycinnamoyl-CoA: shikimate hydroxycinnamoyl transferase (HCT) gene family in poplar
Source: PeerJ. 2021 Feb 25;9:e10741. doi: 10.7717/peerj.10741 (PMC7916539; doi:10.7717/peerj.10741)
Supplement: Supplemental Information 5 [file peerj-09-10741-s005.docx]

The microarray dataset has been submitted in NCBI (GSE56023) and the MIAME has also been provided to NCBI (see the file “popular-tissues-seasons_affy_CHP.xls”.MIAME checklist is as followed:

| Title | Expression data from different tissues in different seasons from P. tomentosa |
| --- | --- |
| Platform organism | [Populus sp.](https://www.ncbi.nlm.nih.gov/Taxonomy/Browser/wwwtax.cgi?mode=Info&id=3697) |
| Sample organism | [Populus tomentosa](https://www.ncbi.nlm.nih.gov/Taxonomy/Browser/wwwtax.cgi?mode=Info&id=118781) |
| Experiment type | Expression profiling by array |
| Experiment description | Different fuctional genes have different expression levels in various tissues. Along with the seasons changing, expression level also changes. One-year expression level for functional genes can be indicated by the four seasonal samples and respective time-course change can also be detected. We use microarry chips to study the expression levels for specific genes and explore new functional genes involved in secondary metabolism |
| Array design | This array was designed based on content from UniGene Build #6 (March 16, 2005) and from GenBank mRNAs and ESTs for all Populus species up to April 26, 2005. Additional array design content was derived from the predicted gene set v1.1 from the Populus genome project (P. trichocarpa), led by the U.S. Department of Energy and based at the Joint Genome Institute (JGI), Walnut Creek, CA (download date May 4, 2005). |
| samples | Samples from four tissues (bud, root, xylem and phloem) were collected in different seasons in one year. April and May were the months in spring for collecting samples. June and July were the months in summer for collecting samples. September and October were the months in autumn for collecting samples. December was the month for collecting samples in winter. RNA from each sample was extracted for hybridization.   \| [GSM1350532](https://www.ncbi.nlm.nih.gov/geo/query/acc.cgi?acc=GSM1350532) \| xylem in spring,biological rep1 \| \| --- \| --- \| \| [GSM1350533](https://www.ncbi.nlm.nih.gov/geo/query/acc.cgi?acc=GSM1350533) \| xylem in spring,biological rep2 \| \| [GSM1350534](https://www.ncbi.nlm.nih.gov/geo/query/acc.cgi?acc=GSM1350534) \| xylem in summer,biological rep1 \| \| [GSM1350535](https://www.ncbi.nlm.nih.gov/geo/query/acc.cgi?acc=GSM1350535) \| xylem in summer,biological rep2 \| \| \| [GSM1350536](https://www.ncbi.nlm.nih.gov/geo/query/acc.cgi?acc=GSM1350536) \| xylem in summer,biological rep3 \| \| \| [GSM1350537](https://www.ncbi.nlm.nih.gov/geo/query/acc.cgi?acc=GSM1350537) \| xylem in fall,biological rep1 \| \| \| [GSM1350538](https://www.ncbi.nlm.nih.gov/geo/query/acc.cgi?acc=GSM1350538) \| xylem in fall,biological rep2 \| \| \| [GSM1350539](https://www.ncbi.nlm.nih.gov/geo/query/acc.cgi?acc=GSM1350539) \| xylem in fall,biological rep3 \| \| \| [GSM1350540](https://www.ncbi.nlm.nih.gov/geo/query/acc.cgi?acc=GSM1350540) \| xylem in winter, biological rep1 \| \| \| [GSM1350541](https://www.ncbi.nlm.nih.gov/geo/query/acc.cgi?acc=GSM1350541) \| xylem in winter, biological rep2 \| \| \| [GSM1350542](https://www.ncbi.nlm.nih.gov/geo/query/acc.cgi?acc=GSM1350542) \| xylem in winter, biological rep3 \| \| \| [GSM1350543](https://www.ncbi.nlm.nih.gov/geo/query/acc.cgi?acc=GSM1350543) \| bud in spring,biological rep1 \| \| \| [GSM1350544](https://www.ncbi.nlm.nih.gov/geo/query/acc.cgi?acc=GSM1350544) \| bud in spring,biological rep2 \| \| \| [GSM1350545](https://www.ncbi.nlm.nih.gov/geo/query/acc.cgi?acc=GSM1350545) \| bud in spring,biological rep3 \| \| \| [GSM1350546](https://www.ncbi.nlm.nih.gov/geo/query/acc.cgi?acc=GSM1350546) \| bud in summer,biological rep1 \| \| \| [GSM1350547](https://www.ncbi.nlm.nih.gov/geo/query/acc.cgi?acc=GSM1350547) \| bud in summer,biological rep2 \| \| \| [GSM1350548](https://www.ncbi.nlm.nih.gov/geo/query/acc.cgi?acc=GSM1350548) \| bud in summer,biological rep3 \| \| \| [GSM1350549](https://www.ncbi.nlm.nih.gov/geo/query/acc.cgi?acc=GSM1350549) \| bud in fall, biological rep1 \| \| \| [GSM1350550](https://www.ncbi.nlm.nih.gov/geo/query/acc.cgi?acc=GSM1350550) \| bud in fall, biological rep2 \| \| \| [GSM1350551](https://www.ncbi.nlm.nih.gov/geo/query/acc.cgi?acc=GSM1350551) \| bud in fall, biological rep3 \| \| \| [GSM1350552](https://www.ncbi.nlm.nih.gov/geo/query/acc.cgi?acc=GSM1350552) \| bud in winter, biological rep1 \| \| \| [GSM1350553](https://www.ncbi.nlm.nih.gov/geo/query/acc.cgi?acc=GSM1350553) \| bud in winter, biological rep2 \| \| \| [GSM1350554](https://www.ncbi.nlm.nih.gov/geo/query/acc.cgi?acc=GSM1350554) \| bud in winter, biological rep3 \| \| \| [GSM1350555](https://www.ncbi.nlm.nih.gov/geo/query/acc.cgi?acc=GSM1350555) \| root in spring,biological rep1 \| \| \| [GSM1350556](https://www.ncbi.nlm.nih.gov/geo/query/acc.cgi?acc=GSM1350556) \| root in spring,biological rep2 \| \| \| [GSM1350557](https://www.ncbi.nlm.nih.gov/geo/query/acc.cgi?acc=GSM1350557) \| root in spring,biological rep3 \| \| \| [GSM1350558](https://www.ncbi.nlm.nih.gov/geo/query/acc.cgi?acc=GSM1350558) \| root in summer, biological rep1 \| \| \| [GSM1350559](https://www.ncbi.nlm.nih.gov/geo/query/acc.cgi?acc=GSM1350559) \| root in summer, biological rep2 \| \| \| [GSM1350560](https://www.ncbi.nlm.nih.gov/geo/query/acc.cgi?acc=GSM1350560) \| root in summer, biological rep3 \| \| \| [GSM1350561](https://www.ncbi.nlm.nih.gov/geo/query/acc.cgi?acc=GSM1350561) \| root in fall, biological rep2 \| \| \| [GSM1350562](https://www.ncbi.nlm.nih.gov/geo/query/acc.cgi?acc=GSM1350562) \| root in fall, biological rep3 \| \| \| [GSM1350563](https://www.ncbi.nlm.nih.gov/geo/query/acc.cgi?acc=GSM1350563) \| root in winter, biological rep1 \| \| \| [GSM1350564](https://www.ncbi.nlm.nih.gov/geo/query/acc.cgi?acc=GSM1350564) \| root in winter, biological rep2 \| \| \| [GSM1350565](https://www.ncbi.nlm.nih.gov/geo/query/acc.cgi?acc=GSM1350565) \| root in winter, biological rep3 \| \| \| [GSM1350566](https://www.ncbi.nlm.nih.gov/geo/query/acc.cgi?acc=GSM1350566) \| phloem in spring, biological rep1 \| \| \| [GSM1350567](https://www.ncbi.nlm.nih.gov/geo/query/acc.cgi?acc=GSM1350567) \| phloem in spring, biological rep2 \| \| \| [GSM1350568](https://www.ncbi.nlm.nih.gov/geo/query/acc.cgi?acc=GSM1350568) \| phloem in spring, biological rep3 \| \| \| [GSM1350569](https://www.ncbi.nlm.nih.gov/geo/query/acc.cgi?acc=GSM1350569) \| phloem in summer, biological rep1 \| \| \| [GSM1350570](https://www.ncbi.nlm.nih.gov/geo/query/acc.cgi?acc=GSM1350570) \| phloem in summer, biological rep2 \| \| \| [GSM1350571](https://www.ncbi.nlm.nih.gov/geo/query/acc.cgi?acc=GSM1350571) \| phloem in summer, biological rep3 \| \| \| [GSM1350572](https://www.ncbi.nlm.nih.gov/geo/query/acc.cgi?acc=GSM1350572) \| phleom in fall,biological rep1 \| \| \| [GSM1350573](https://www.ncbi.nlm.nih.gov/geo/query/acc.cgi?acc=GSM1350573) \| phleom in fall,biological rep2 \| \| \| [GSM1350574](https://www.ncbi.nlm.nih.gov/geo/query/acc.cgi?acc=GSM1350574) \| phleom in winter, biological rep1 \| \| \| [GSM1350575](https://www.ncbi.nlm.nih.gov/geo/query/acc.cgi?acc=GSM1350575) \| phleom in winter, biological rep2 \| \| \| [GSM1350576](https://www.ncbi.nlm.nih.gov/geo/query/acc.cgi?acc=GSM1350576) \| phleom in winter, biological rep3 \| \| |

PROTOCOLS (Measurements Normalization controls)

growth protocol Five–year-old populus tomentosa 741 strains in field were harvested as plant material in this research from Hebei, China

treatment protocol Samples collected were immediately frozen in liquid nitrogen and then stored in -80℃ until use.

extract protocol Trizol extraction of total RNA was performed according to the manufacturer's instructions.

label protocol cRNA were prepared according to the standard Affymetrix protocol from 6 ug total RNA (Expression Analysis Technical Manual, 2001, Affymetrix).

hyb protocol Following fragmentation, 10 ug of cDNAwere hybridized for 16 hr at 45C on GeneChip Drosophila Genome Array. GeneChips were washed and stained in the Affymetrix Fluidics Station 400.

scan protocol GeneChips were scanned using the GeneChip® Scanner 3000.

data processing The data were analyzed with Microarray Suite version5.0 (MAS 5.0) using Affymetrix default analysis settings and global scaling as normalization method. The trimmed mean target intensity of each array was arbitrarily set to 100.
